# Experience of maternal immunization among women living with HIV in Belgium: A mixed-methods study^☆^

**DOI:** 10.1016/j.pmedr.2025.103107

**Published:** 2025-05-10

**Authors:** Yama Touré, Charlotte Martin, Coca Necsoi, Marc Delforge, Déborah Konopnicki, Nicolas Dauby

**Affiliations:** a Department of Infectious Diseases, CHU Saint-Pierre, Université Libre de Bruxelles (ULB), Brussels, Belgium; b Centre for Environmental Health and Occupational Health, School of Public Health, Université Libre de Bruxelles (ULB), Brussels, Belgium

**Keywords:** Maternal immunization, Pregnancy, HIV-exposed uninfected, Influenza, COVID-19, Vaccine hesitancy, Vaccination

## Abstract

**Objective:**

Maternal immunization during pregnancy has benefits for both women living with HIV (WLWH) and their HIV-exposed uninfected newborns. Vaccine hesitancy may hamper vaccine acceptability during pregnancy. We aimed to assess the experience of WLWH with a history of pregnancy with maternal immunization.

**Methods:**

In-person quantitative survey with embedded collection of qualitative data was performed among WLWH with a history of pregnancy**.**

**Results:**

Between October and November 2021, a total of 20 WLWH followed at Saint-Pierre University Hospital Center HIV reference center in Brussels (Belgium) were included. The majority originated from Sub-Saharan Africa and had education level below or equivalent to secondary school. Most of the participants agreed that vaccination during pregnancy is necessary, highlighting the benefits for the health of the mother and the newborn. Reluctant participants expressed concerns about safety or lack of information. Most of the participants mentioned health-care providers (HCP) (mainly HIV physician) as the main person of trust for vaccine information in general. Social media were also mentioned as an important source of information on vaccination.

**Conclusions:**

The benefits for the mother and the newborn were associated with favorable opinion on maternal immunization while doubt about safety or lack of information appeared to be hurdles to acceptance. Proactive communication on the benefit of maternal immunization for both the mother and newborn by HIV physician and HCP involved in prenatal care is needed to increase acceptability of maternal immunization among WLWH.

## Highlights

• Vaccine hesitancy may hamper the acceptability of maternal immunization.
• A study among women living with HIV in Belgium.
• Methods include in-person quantitative survey with collection of qualitative data.
• Most of the participants agreed that vaccination during pregnancy is necessary.
• Hesitancy was driven by concerns about safety or lack of information.

## 1 Introduction

According to the Strategic Advisory Group of Experts on Immunization working group on Vaccine Hesitancy, vaccine hesitancy refers to the delay in acceptance or refusal of vaccination despite the availability of vaccination services (MacDonald, 2015). It is considered one of the top 10 threats to global health (WHO, 2019). Rather than a static behavior toward vaccination, vaccine hesitancy is now considered as a “state of indecisiveness regarding a vaccination decision” (Bussink-Voorend et al., 2022). It is a dynamic process that may be mitigated by interventions such as motivational interview (Gagneur, 2020).

Maternal immunization is currently a well-established strategy to decrease the burden of different infections with increased severity during pregnancy and/or the post-natal period (Dauby et al., 2024).

Maternal immunization is of special interest for the health of women living with HIV (WLWH) but also of their HIV-exposed uninfected infants, who are at increased risk of certain post-natal vaccine-preventable diseases such as respiratory syncytial virus, influenza or whooping cough. Routinely recommended vaccines during pregnancy include vaccines against *Bordetella pertussis* (Tetanus-Diphtheria-acellular Pertussis vaccine), respiratory syncytial virus, influenza and COVID-19 (Dauby et al., 2024). The majority of WLWH in Europe are migrants originating from Sub-Saharan Africa, especially among the newly diagnosed patients (Owusu et al., 2023). Different studies mostly performed during the COVID-19 pandemic found that women of African descent were more likely to be vaccine hesitant (Abba-Aji et al., 2022). Limited evidence suggests that WLWH might be less vaccinated compared to HIV-uninfected pregnant women. In a study performed in the United States of America, WLWH were less likely to be vaccinated during pregnancy after adjustment for insurance, comorbidities, age and parity; the reasons were however not explored (Doraivelu et al., 2019).

Different predictors of vaccine uptake during pregnancy have already been identified and include recommendation by health care providers, disease risk perception and vaccine awareness. However, all studies have been performed in the general HIV-uninfected population (Mendez et al., 2025; Skjefte et al., 2021; Karafillakis et al., 2021). No study has specifically assessed vaccine acceptance among WLWH. Given the vulnerability of WLWH in Europe due to different factors (migration, ethnicity and gender) and the possible impact on their health and of their newborns, it is relevant to identify barriers to maternal immunization in this specific population.

We aimed to assess the experience of WLWH with maternal immunization and identify factors associated with acceptance or refusal of vaccines during a previous pregnancy.

## 2 Material and methods

Between October and November 2021, all women aged between 18 and 50 years with a history of pregnancy in the last 10 years and in active follow-up at the HIV reference center of Saint-Pierre University Hospital Center, Brussels were identified using the HIV reference center's database before their visit to the outpatient clinic or the clinical research unit. On the day of their appointment while the patient was waiting for the physician's consultation, a research nurse (Y.T.) approached the women in the waiting room and invited them to participate in the study. Of the 31 women who were invited to participate in the study, a total of 20 women accepted.

For data collection, we used a mixed methods approach with a predominant quantitative method. A quantitative study using an in-person paper-based survey was performed to assess the participants' attitude toward health decisions and vaccination, both in general and during pregnancy. The survey was developed in collaboration with nurses, social workers, and psychologists working at the outpatient clinic both in French and English languages. Qualitative themes were embedded within the quantitative outcomes to better explain the participants' responses (Kajamaa et al., 2020). No semi-structured interview was used; we collected the participants' comments on paper while answering the survey. Besides the collection of verbal data, the qualitative approach allowed us to explore non-verbal gestures and patients' feelings but also personal experiences. The survey was completed in the presence of the research nurse trained in qualitative methods. It was estimated that the completion of the survey would require approximately 30 min for each participant. Detailed answers, including quotes were collected on paper during the interviews and subsequently entered in a REDCap form hosted at University Hospital Center Saint-Pierre (Harris et al., 2009). HIV-infection related data (viral load and CD4 count) were extracted from the medical files and entered in a Redcap form. Only descriptive statistical analyses were used and were performed with Microsoft Excel. The study was approved by the local ethics committee of University Hospital Center Saint-Pierre (CE/210913) and all participants signed informed consent form. Confidentiality of participants ‘data was preserved in accordance with the General Data Protection Regulation. All data collected from participants were anonymized. Furthermore, the study protocol was reviewed and approved by the Data Protection Officer at University Hospital Center Saint-Pierre, ensuring compliance with ethical and legal standards for data protection.

## 3 Results

The characteristics of the women included in the study are summarized in Table 1. There were no differences in terms of ethnicity, CD4 count, and viral load between the participants in the study and WLWH followed in our cohort who met the same inclusion criteria. The majority (90 %) were French-speaking, and the remainder were English-speaking. A substantial proportion (70 %) had a precarious social situation, with a high proportion being asylum seekers and/or undocumented. Most of the women (65 %) reported an education level below or equivalent to secondary school. All women reported healthcare providers as an important source of information for vaccination in general; the HIV physician was the most trusted person followed by the general practitioner. It should be noted that only 7 % of the participants were confident with a nurse providing information about vaccines. Social media was reported as an important source of information on vaccination by a high proportion of the participants (60 %).

**Table 1:** Descriptive statistics of the socio-demographic and HIV-infection characteristics of women living with HIV in Belgium and their source of information about vaccination, october–november 2021.

Characteristic	N (%)
Age, median (IQR) (years)	37 (23–48)

Country of Birth	
Sub-Saharan Africa	17 (85)

Religion	
Catholic	12 (60)
Muslim	5 (2)
No religion	2 (10)
Prefer not to respond	1 (5)

Level of education	
Primary school	2 (10
Secondary school	11 (55)
Higher education	3 (15)
University	3 (15)
Prefer not to respond	1 (5)

Source of income	
Declared work	6 (30)
Unemployment	6 (30)
Income support	4 (20)
Work disability	1 (5)
Other	3 (15)
Viral load <50 copies/ml	20 (100)
CD4 T cell count (IQR)	638 (536–832)

About vaccination information, who do you trust? (several answers allowed)	
Health care provider	20 (100)
HIV Physician	16 (78)
General practitioner	11 (56)
Gynecologist	4 (22)
Nurse	2 (7)
Partner / Husband	3 (11)
Other member of the family	3 (11)
Social networks	12 (60)

*#* Countries represented were Cameroun, Ivory Coast, Democratic Republic of the Congo, Gabon, Guadeloupe, Republic of Guinea, Niger, Czech Republic, Rwanda, Syria, Zambia.

IQR: interquartile range.

Results regarding the attitude toward health decision and experiences of the women with maternal immunization are summarized in Table 2, along with illustrative quotes collected during the interviews. While most participants discussed health issues with their partner or a family member, only a minority acknowledged the critical role that these individuals play in health-related decision making. Of note, most participants agreed that vaccination is necessary during pregnancy. The vaccine most frequently administered during pregnancy was the influenza vaccine, followed by COVID-19 and the Tetanus-Diphtheria-acellular pertussis vaccine. The reasons given by these participants mainly related to the importance of protecting the mother and the newborn:

**Table 2:** Attitude toward health decisions, feelings and experience with maternal immunization along with quotes collected during qualitative assessment among women living with HIV in Belgium, october–november 2021.

	Answer N (%)	Exemplary quote	Answer N (%)	Exemplary quote
Do you talk about health issues with someone from your household or family?	Yes 16(79) Partner husband 13 (67) Family member other than husband 10 (53) Friend 1 (5) Religious authority 3 (15) Healer 0 (0)			
Does this person have critical advice on your decision regarding your health⁎	Yes 6 (30)	“*It is a couple decision.*” Age 32-Rwanda “*Because he is my partner and father of my daughter.*” Age 38-Cameroon “*It is my husband who brought me in Europe.*” Age 37-Guinea	No 7 (35)	“*The initiative is personal.”* Age 25*-Guinea* *“This person leaves it up to me to* *to decide.”* Age 29*-Guinea* *“I make my own decisions.”* Age 49*-Zambia* *“Because it's about my health.”* Age 41*-Cameroon* *“The person has his or her point of view, but* *I have the last word.”* Age 37*-Democratic Republic of the Congo* *“My health is personal.”* Age 46*-Ivory Coast* *“I'm enough informed to make my own* *decisions.”* Age 39*-Rwanda* *“Any decision depends on me.”* Age 47*-Cameroon*
Do you think that immunization during pregnancy is needed?	Yes 13 (65)	“*Immunization protects the mother and the baby.”* Age 37*-Gabon* *“To help your pregnancy run successfully.”* Age 41*-Cameroon* *“If it could help me and the baby.”* Age 49*-Zambia* *“To protect the baby and the mom”* Age 38*-Cameroun* *“To protect the child and myself, all mothers do that”* Age 34*-Guinea* *“Probably needed following medical advice”* Age 47*-Cameroun* *“Protect myself and the baby too” #10-Democratic Republic of the Congo*	No 7(35)	*“Fear of problems with the baby.”* Age 39*-Rwanda* *“I do not know the effects and it doesn't prevent disease.”* Age 29*-Guinea* *“Not enough information on vaccination to decide.”* Age 37*-Guinea*
Did you accept a vaccine during your last pregnancy?	15 (75)		5 (25)	I fear vaccine 7(35) I feel that I have not received appropriate information 13 (65)

⁎ : Prefer not to respond 7 (35)

“*Immunization protects the mother and the baby.”* Age 37*-Gabon.*

*“To help your pregnancy run successfully.”* Age 41*-Cameroon.*

The reasons given by the participants who disagree with the fact that vaccination is necessary during pregnancy were related to safety issues and lack of knowledge about the possible benefits/risks:

*“Fear of problems with the baby.”* Age 39*-Rwanda.*

*“I do not know the effects, and it doesn't prevent disease.”* Age 29*-Guinea.*

*“Not enough information on vaccination to decide.”* Age 37*-Guinea.*

Among the women who accepted vaccination during pregnancy, the majority (60 %) made the decision independently, based on personal beliefs while a minority decided after a discussion with healthcare providers, mostly gynecologists:

*“I make my own decisions.”* Age 49*-Zambia.*

*“The person has his or her point of view, but I have the last word.”* Age 37*-Democratic Republic of the Congo.*

## 4 Discussion

In the present study, we assessed the acceptance and experience of a small group of WLWH living in Belgium with maternal immunization using mixed methods with a predominantly quantitative method. Our findings indicate that the majority of the participants had positive feelings about maternal immunization, as highlighted by qualitative data collected during interviews.

Women with positive experience with maternal immunization mostly highlighted the benefits for both the mother and the infants. An interventional study in the general population found that communication about the risks of the disease preventable by vaccination was the most effective strategy as compared to interventions aimed at undercutting vaccination myths (Horne et al., 2015).

Most of the women included in the study originated from Sub-Saharan Africa with an important proportion suffering from social precariousness and low educational level as previously reported (Owusu et al., 2023; Gobert et al., 2021). Migration status has been shown to impact vaccine coverage among people living with HIV (Gobert et al., 2021; Grabmeier-Pfistershammer et al., 2015). Independently of HIV status, migration is associated with higher risk of vaccine hesitancy, mostly related to concerns about vaccine safety and distrust in the healthcare system (Tankwanchi et al., 2021). Different research in Europe have found lower rate of vaccine acceptance during pregnancy among women of African descent (Davies et al., 2022). As reported by the participants of the present study, the main reasons for refusing vaccination during pregnancy were concerns about vaccine safety and/or lack of information.

Our findings also stress the important role of information by healthcare providers in the acceptance of maternal immunization: the HIV physician was the most trusted source of information regarding vaccination in general. These results are consistent with larger studies that identified the recommendations from an healthcare providers as the strongest predictor of vaccine uptake during pregnancy (Karafillakis et al., 2021). Recommendations for maternal immunization for WLWH should be part of routine HIV care and should not only rely on gynecologists or other healthcare providers involved in prenatal care. Importantly, the European AIDS Clinical Society guidelines do not mention the importance of maternal immunization despite clear evidence of its benefits (Dauby et al., 2024), which were also the main reason reported by mothers in this study. Pediatricians may be involved in the antepartum care of WLWH providing counseling about infant feeding and antiretroviral prophylaxis. Although we did not explore the role of pediatricians in vaccine acceptability during pregnancy, they could represent an important source of information about maternal immunization.

Our study has some limitations. First, the sample size was small. However, it provides a representative sample of WLWH in care in Belgium, i.e. high proportion of women from Sub-Saharan Africa with low educational levels. This population is difficult to reach using online or electronic questionnaires, which are usually used in vaccine acceptability studies (Skjefte et al., 2021; Davies et al., 2022); thus the use of mixed-method with in-person interviews could be considered a strength of the study. Secondly, although qualitative themes were collected, illiterate women could not participate due to the requirement of an informed consent form.

In conclusion, proactive communication by HIV physicians in collaboration with healthcare providers involved in prenatal care on the benefits of maternal immunization for both the mother and newborn, should be part of routine care of WLWH during pregnancy and could likely contribute to increase acceptability of the different vaccines recommended during pregnancy. The results from this pilot study, despite the limited number of participants, can inform the development of a protocol for a larger multicentric study by highlighting key variables and potential methodologies.

## Support for the reported work

none.

## Outside this work

N.D. has received a grant from MSD Belgium.

## CRediT authorship contribution statement

**Yama Touré:** Writing – review & editing, Writing – original draft, Investigation, Formal analysis, Data curation, Conceptualization. **Charlotte Martin:** Writing – review & editing, Methodology, Conceptualization. **Coca Necsoi:** Writing – review & editing, Resources, Formal analysis, Data curation. **Marc Delforge:** Software, Resources, Investigation, Formal analysis, Data curation. **Déborah Konopnicki:** Writing – review & editing, Methodology, Investigation, Data curation, Conceptualization. **Nicolas Dauby:** Writing – review & editing, Writing – original draft, Supervision, Formal analysis.

## Declaration of competing interest

The authors declare that they have no known competing financial interests or personal relationships that could have appeared to influence the work reported in this paper.

## Data Availability

Data will be made available on request.
